# An Analysis of French-Language Tweets About COVID-19 Vaccines: Supervised Learning Approach

**DOI:** 10.2196/37831

**Published:** 2022-05-17

**Authors:** Romy Sauvayre, Jessica Vernier, Cédric Chauvière

**Affiliations:** 1 Laboratoire de Psychologie Sociale et Cognitive Université Clermont Auvergne Centre national de la recherche scientifique Clermont-Ferrand France; 2 Polytech Clermont Clermont Auvergne INP Aubiere France; 3 Laboratoire de Mathématiques Blaise Pascal Université Clermont Auvergne Centre national de la recherche scientifique Clermont-Ferrand France

**Keywords:** social media, natural language processing, public health, vaccine, machine learning, CamemBERT language model, method, epistemology, COVID-19, disinformation, language model

## Abstract

**Background:**

As the COVID-19 pandemic progressed, disinformation, fake news, and conspiracy theories spread through many parts of society. However, the disinformation spreading through social media is, according to the literature, one of the causes of increased COVID-19 vaccine hesitancy. In this context, the analysis of social media posts is particularly important, but the large amount of data exchanged on social media platforms requires specific methods. This is why machine learning and natural language processing models are increasingly applied to social media data.

**Objective:**

The aim of this study is to examine the capability of the CamemBERT French-language model to faithfully predict the elaborated categories, with the knowledge that tweets about vaccination are often ambiguous, sarcastic, or irrelevant to the studied topic.

**Methods:**

A total of 901,908 unique French-language tweets related to vaccination published between July 12, 2021, and August 11, 2021, were extracted using Twitter’s application programming interface (version 2; Twitter Inc). Approximately 2000 randomly selected tweets were labeled with 2 types of categorizations: (1) arguments for (pros) or against (cons) vaccination (health measures included) and (2) type of content (scientific, political, social, or vaccination status). The CamemBERT model was fine-tuned and tested for the classification of French-language tweets. The model’s performance was assessed by computing the F1-score, and confusion matrices were obtained.

**Results:**

The accuracy of the applied machine learning reached up to 70.6% for the first classification (pro and con tweets) and up to 90% for the second classification (scientific and political tweets). Furthermore, a tweet was 1.86 times more likely to be incorrectly classified by the model if it contained fewer than 170 characters (odds ratio 1.86; 95% CI 1.20-2.86).

**Conclusions:**

The accuracy of the model is affected by the classification chosen and the topic of the message examined. When the vaccine debate is jostled by contested political decisions, tweet content becomes so heterogeneous that the accuracy of the model drops for less differentiated classes. However, our tests showed that it is possible to improve the accuracy by selecting tweets using a new method based on tweet length.

## Introduction

### Background

The COVID-19 pandemic has profoundly affected our society and social activity worldwide. Part of this change is perceptible through messages exchanged on social media platforms, specifically on the topic of vaccination. Since the measles, mumps, and rubella vaccine controversy in 1998 [1], vaccine hesitancy has grown on the internet [2,3] and subsequently on social media platforms such as Facebook and Twitter [4,5]. In the same way, as the pandemic progressed, disinformation, “fake news,” and conspiracy theories spread [6] through many parts of society. However, the disinformation spreading through social media is, according to the literature, “potentially dangerous” [7] and is one of the causes of increased COVID-19 vaccine hesitancy [8,9]. Another cause mentioned in the literature is the loss of confidence in science among the public [10].

In this context, social media analysis is particularly important, but the large amount of data exchanged over social networks requires specific methods. This is why machine learning and natural language processing (NLP) models are becoming increasingly popular for studying social media data. The most used and “most promising method” [11] is sentiment analysis. For example, sentiment analyses were conducted on messages posted on Twitter (tweets) to measure the opinions of Americans regarding vaccines [12] and evaluate the rate of hate tweets among Arab people [13]. Additionally, another method, opinion mining, is used and has obtained an equal level of maturity [14]. Both methods attempt to identify and categorize subjective content in text, but it is not an easy task to correctly identify such concepts (opinion, rumor, idea, claim, argument, emotion, sentiment, and affect). The fields of psychology and philosophy have extensively studied these concepts but have raised the difficulty of defining their boundaries. This is why stance detection has grown to be considered “a subproblem of sentiment analysis” [15]. In addition, according to Visweswaran et al [16], performing a sentiment analysis on tweets is a challenge because tweets contain short text (280 characters or less), abbreviations, and slang terms. However, few studies focus on the difficulties encountered by a neural network according to the chosen categories [17]. The aim of this paper is to provide additional methodological reflection.

### Objective

The aim of this study is to examine the capability of the CamemBERT model to faithfully predict the elaborated categories while considering that tweets about vaccination are often ambiguous, sarcastic, or irrelevant to the studied topic. Based on the resulting analysis, this paper aims to provide a methodological and epistemological reflection on the analysis of French-language tweets related to vaccination.

### A State-of-the-art French-Language Model

The CamemBERT model was released in 2020 and is considered one of the state-of-the-art French-language models [18] (together with its close “cousin” flauBERT [19]). It makes use of the Robustly Optimized BERT Pretraining Approach architecture of Liu et al [20], which is an improved variant of the famous Bidirectional Encoder Representations From Transformers (BERT) architecture of Devlin et al [21]. The BERT family of models consists of general, multipurpose, pretrained models that may be used for different NLP tasks, including the following: classification, question answering, and translation. They rely heavily upon transformers, which have radically changed the performance of NLP tasks since their introduction by Google researchers in 2017 [22]. They have been pretrained on a large corpus ranging from gigabits to terabits of data, using considerable computing resources.

Although multilingual models are plentiful, they usually lag behind their monolingual counterparts. This is why, in this study, we chose to employ a monolingual model to classify French-language tweets. As far as we are concerned, CamemBERT comes in 6 different “flavors,” ranging from small models with 110 million parameters trained on 4 GB of text up to mid-size models with 335 million parameters trained on 135 GB of text. After testing them, we found that better results were obtained with the largest size model that was pretrained on the Criss-Cross Network corpus.

All these models require fine-tuning on specific data to achieve their full potential. Fine-tuning or transfer learning have been common and successful practices in computer vision for a long time, but it is only in the last 3 years or so that the same approaches have become effective for solving NLP problems on specific data. This approach can be summarized in the following 3 steps:

1. A model language such as BERT is built in an unsupervised manner using a large database, removing the need to label data.
2. A specific head (such as dense neural network layers) is added to the previous model to make it task-specific.
3. The new model is trained in its entirety with a small learning rate on specific data.

The first step is usually performed by large companies, such as Google or Facebook, or public research centers that make their model freely available on internet platforms. The second and third steps form a process that is generally referred to as *fine-tuning*, and this is what we will do in this study.

## Methods

### Data Collection

**Figure 1:**
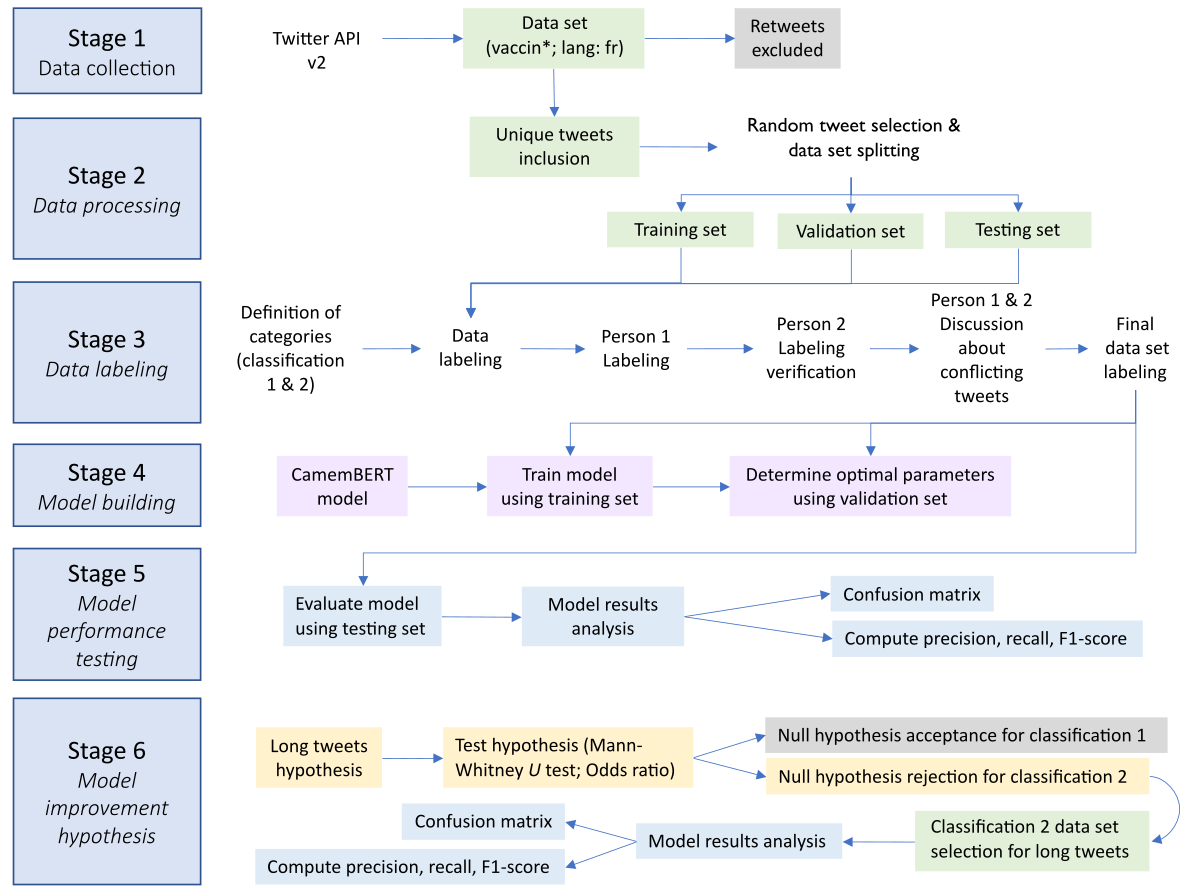
Flow chart of methodology steps. API v2: application programming interface version 2.

French-language tweets published between July 12, 2021, and August 11, 2021, were extracted using the Twitter application programming interface ([API] version 2; Twitter Inc; Figure 1) with a Python (Python Software Foundation) script request (vaccin lang: fr), and several elements (tweet content, tweet ID, author ID, and creation date) were stored in a document-oriented database (MongoDB, MongoDB Inc). As queries can only contain a limited number of terms (1024 characters), it was more relevant to search for the word *vaccin* (“vaccine”), knowing that related terms were included by the Twitter API version 2 search tools since November 15, 2021, rather than selecting a nonexhaustive keyword list. Indeed, Twitter’s query tool collected all words containing the base word *vaccin* in French (ie, *vaccin*, *vaccins*, *vaccination*, *vaccinations*, *vaccinat*, *vacciner*, *vaccinés*, *vaccinées*, *vaccinerait*, *vaccineraient*, *pro-vaccin*, *anti-vaccin*, *#vaccin*, *#vaccinationobligatoire*). The goal of this approach was to collect all tweets containing the base word *vaccin* to explore their content using a bottom-up approach without additional inclusion or exclusion criteria. A total of 1,782,176 tweets were obtained, including 901,908 unique tweets (29,094 tweets per day) published by 231,373 unique users. To fully test the CamemBERT model, only unique tweets were included in the analysis. When dealing with the analysis of text (such as tweets), it is important to keep a large amount of variability (eg, vocabulary, syntax, and length) to strengthen deep learning algorithms. This variability will guarantee the power of model generalization. This is why, in this study, the 1851 tweets that comprise the data set were drawn randomly from a set of 901,908 unique tweets.

### Labeling

A total of 1851 unique tweets were randomly selected and manually labeled by 2 people (1451 for training and validation and 400 for testing). When doubt arose about labeling, which occurred for 87 of the 1851 tweets (4.7%), a discussion occurred to determine the relevant label for each tweet (see examples in Multimedia Appendix 1). Note that no duplicates were identified by the automated verification performed.

A total of 2 classifications were developed to examine arguments for (pros) or against (cons) vaccination (health measures included) and examine the type of tweet content (scientific, political, social, or vaccination status). The classifications and definitions used to label tweets are provided in Table 1 with translated examples of tweets for each label. In accordance with Twitter's terms of use under the European General Data Protection Regulation, original tweets cannot be shared [23]. Therefore, the translations have been adjusted to ensure the anonymity of Twitter users.

**Table 1:** Classification criteria for tweets and definitions.

Type of tweet		Definition	Translated examples (French to English)
**Classification problem 1**			
	Unclassifiable	Unclassifiable or irrelevant to the topics of vaccination or health measures	The Emmanuel Macron effect
	Noncommittal	Neutral or without explicit opinion on vaccination and/or the health pass	I have to ask my doctor for the vaccine
	Pros	Arguments in favor of the health pass Arguments in favor of the COVID-19 vaccine and/or the health pass (efficiency, safety, relevance)	Personally, I am vaccinated so nothing to fear, on the other hand, good luck to all the anti-vaccine, you will not have the choice now??
	Cons	Arguments against vaccination or doubts about the effectiveness of COVID-19 vaccines, fear of side effects, and refusal to obtain the health pass	I am against the vaccine I am not afraid of the virus but I am afraid of the vaccine
**Classification problem 2**			
	Unclassifiable	Irrelevant to the topic or unclassifiable	A vaccine
	Scientific	Scientific or pseudoscientific content that uses true beliefs or false information	The vaccine is 95% efficient, a little less in fragile people. The risk is not zero, but a vaccinated person has much less chance of transmitting the virus.
	Political	Comments on legal or political decisions about vaccination or health measures	Basically the vaccine is mandatory, shameful LMAO
	Social	Comments, debates, or opinions on the report to other members of society	“Pro vaccine” you have to also understand that there are people who do not want to be vaccinated.
	Vaccination status	Explicit tweet about the vaccination status of the tweet author Comments on the symptoms experienced after COVID-19 vaccination Explicit refusal to receive a COVID-19 vaccine	Example 1: I am very glad to have already done my 2 doses of the vaccine, fudge Example 2: I don't want to get vaccinated. Why? Well, you know, we don't know what's in this vaccine, it can be dangerous.

### Classification Method

This study followed the general methodology of machine learning to guarantee a rigorous building of the model. To ensure that the model did not overfit or underfit the data set, the following steps were taken:

1. The data set was divided into training (n=1306), validation (n=145), and testing (n=400) data sets.
2. The training loss was represented as a function of the number of epochs to monitor the correct learning of the model and select its optimal value.
3. The validation accuracy is represented as a function of the number of epochs to ensure that the model was not overfitting or underfitting the data.
4. The final model was evaluated on a testing data set that had not been previously used to build or validate the model.

A total of 2 fully connected dense neural network layers with 1024 and 4 neurons (for classification problem 1) or 5 neurons (for classification problem 2) were added to the head of the CamemBERT model, adding another 1.6 million parameters. Furthermore, to prevent overfitting, a 10% dropout was applied between those 2 layers. A small learning rate of 2 × 10^-5^ was used for fine-tuning, and adaptive moment estimation with a decoupled weight decay regularization [24] was chosen as the optimizer (see full code used on GitHub [25]). The parameters were adjusted by minimizing the cross-entropy loss, which is a common choice when dealing with a classification problem. Fine-tuning was performed on a data set consisting of the 1451 labeled French-language tweets, 90% (n=1306) of which were used for training and the remaining 10% (n=145) for validation. Once the model was built, it was tested on a new set of 400 labeled tweets from which a statistical analysis was performed. A total of 2 classification models were built from the same data set, 1 with 4 labels (unclassifiable, neutral, positive, or negative) related to a tweet author’s opinion about vaccination and 1 with 5 labels related to the type of content in a tweet (unclassifiable, scientific, political, social, vaccination status, or symptoms). The proportion of tweets classified into each label for these 2 problems is given in Table 2. We see that the data set is slightly imbalanced. As such, it does not require special treatment.

**Figure 2:**
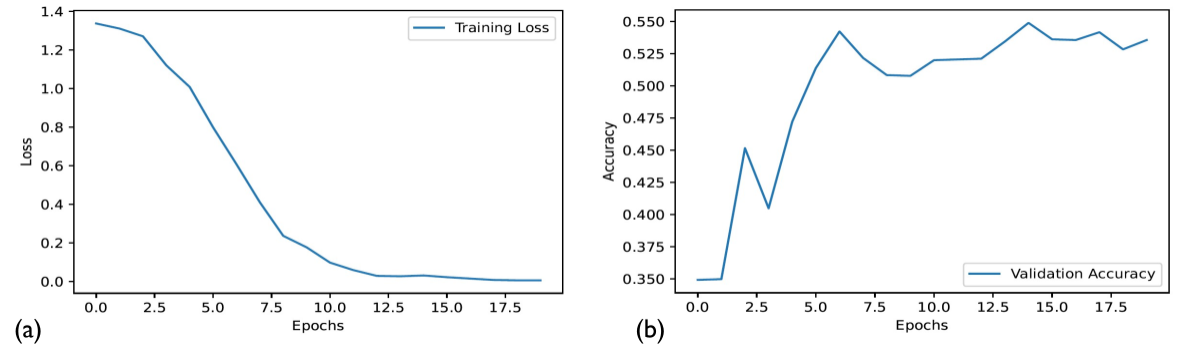
Training loss (a) and validation accuracy (b) of the model over 20 epochs for classification problem 1.

One of the main hyperparameters to be tuned for the training of the model is the number of epochs. As a rule of thumb, to prevent overfitting, the number of epochs is usually chosen based on when the abruptness of the slope of the loss changes while maintaining a low rate of misclassification on the validation data set. Figure 2 shows that 7 epochs should lead to the best result.

This was confirmed by computing the precision, recall, and F1-score at 3 different epochs (7, 15, and 20), as shown in Table 3. The reported results were computed on the test data set with 400 tweets. The average results over the classes were weighted to account for imbalanced classes in the data set. As expected, the highest score was obtained with 7 epochs, however, not by a wide margin (Table 3).

A similar study for the second classification problem determined that 6 epochs were enough to prevent overfitting. The performance of the model was also measured by computing the weighted precision, recall, and F1-score, as shown in Table 4.

The size of the data set is quite similar to those of Kummervold et al [17] (1633 tweets for training and 544 for testing) and Benítez-Andrades et al [26] (n=1400 for training and n=600 for testing). Furthermore, the benefit of using a pretrained model such as the CamemBERT is that a large data set is not required to obtain good results. We also tried to build a neural network model from scratch with the same data set, but the classification performance of the model was significantly lower than the results presented in this paper with the CamemBERT model. For classification problem 1, we reached an accuracy of 33% (versus 59% with the pretrained model) and for classification problem 2, we reached an accuracy of 40% (versus 67.6% with the pretrained model).

**Table 2:** The proportion of tweets assigned to each label in the data set for classification problems 1 and 2 (n=1451).

Classification problem		Tweets
**Classification problem 1, n (%)**		
	Unclassifiable	189 (13)
	Neutral	354 (24.4)
	Positive	392 (27)
	Negative	516 (35.6)
**Classification problem 2, n (%)**		
	Unclassifiable	226 (15.6)
	Scientific	441 (30.4)
	Political	316 (21.8)
	Social	353 (24.3)
	Vaccination status	115 (7.9)

**Table 3:** Classification performance of the model for classification problem 1.

Epochs, n	Precision^a^	Recall^a^	F1-score^a^
7	59	55.3	55.3
15	56.6	53	53.2
20	56.9	54.5	55.2

^a^These data are provided as percentages.

**Table 4:** Classification performance of the model for classification problem 2.

Epochs, n	Precision^a^	Recall^a^	F1-score^a^
6	67.6	64.5	62.9
15	62.7	62.8	61.3
20	60.6	59.5	56.5

^a^These data are provided as percentages.

## Results

### Statistical Analysis

From the results of the previous section, we see that it is significantly more difficult to build a performant classifier based on the 4 vaccine sentiment labels (unclassifiable, noncommittal, pros, and cons), with the maximum F1-score reaching 55.3% in this case. On the other hand, the classifier built from the same tweets but with 5 different labels based on content type (unclassifiable, scientific, political, social, vaccination status, or symptoms) achieved a much higher F1-score (62.9%).

**Figure 3:**
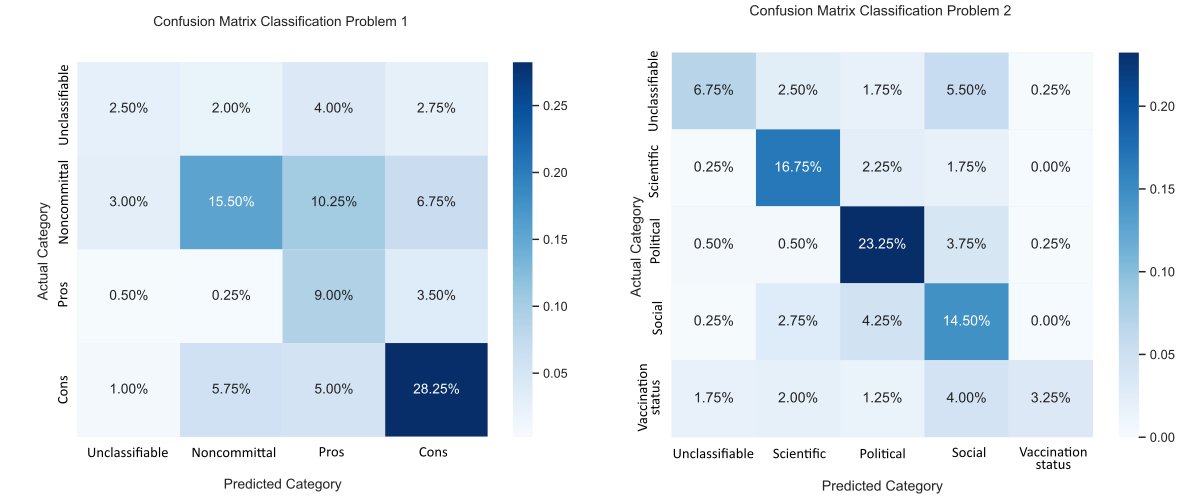
Confusion matrix for classification problems 1 and 2 (n=400).

To analyze the strength and weakness of a model more specifically, it is always instructive to represent it using a confusion matrix [27], as shown in Figure 3.

Since the values in these matrices are percentages, their interpretation requires some care. For the first problem, summing figures line-by-line in the matrix shows that out of 100 tweets from the test data set, on average, 11.25 are unclassifiable, 35.50 are noncommittal, 13.25 are pros, and 40.00 are cons. It is then possible to compute the proportion of tweets correctly classified by the model, label-by-label. The results are shown in Table 5. We see that the model can accurately classify the tweets labelled as pros and cons. It misclassifies a large number of the unclassifiable tweets and, to a lesser extent, noncommittal tweets. Looking back to the confusion matrix, for the last 2 labels, we observe that the model tends to classify the tweets as being pros.

For the second problem, as expected, in line with the higher F1-score found in the previous section, the model achieves much better classification performance. It excels at classifying scientific and political tweets and is also good at classifying social tweets. It still has some difficulties classifying unclassifiable tweets and, in a larger proportion, vaccination status tweets. Looking back to the confusion matrix, for the last 2 labels, we observe that the model tends to classify them as being social tweets.

**Table 5:** The number of tweets correctly classified for each label in classification problems 1 and 2 (n=400).

Classification problem		Tweets
**Classification problem 1, n (%)**		
	Unclassifiable	10 (22.2)
	Noncommittal	62 (43.7)
	Pros	36 (67.9)
	Cons	113 (70.6)
**Classification problem 2, n (%)**		
	Unclassifiable	27 (40.3)
	Scientific	67 (79.8)
	Political	93 (82.3)
	Social	58 (66.7)
	Vaccination status	13 (26.5)

### Text Size Analysis

**Figure 4:**
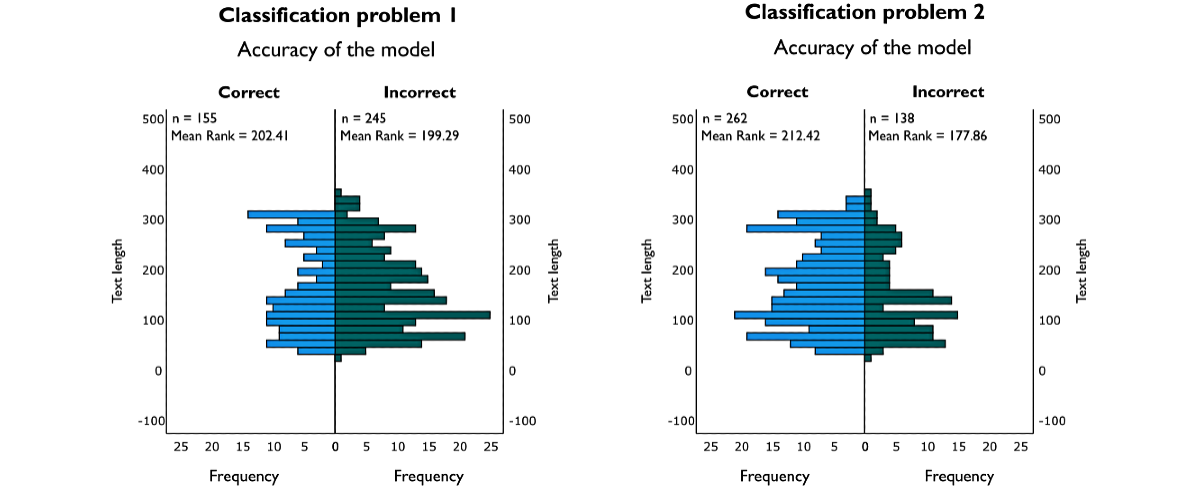
Tweet text length as a function of the accuracy of the fine-tuned CamemBERT model conducted on classification problems 1 and 2 (Mann-Whitney *U* test).

To improve the performance of the fine-tuned CamemBERT model, a hypothesis about the influence of tweet length on model accuracy was tested. A Mann-Whitney *U* test generated statistically significant results for classification problem 2 (*U*=21,202; *P*=.004) but not for classification problem 1 (*U*=19,284; *P*=.79). As Figure 4 shows, the correctly predicted tweets are significantly longer for classification problem 2. A second analysis carried out on a dichotomous variable created from the tweet text length (greater than or less than 170 characters) confirmed this significance for classification problem 2. A tweet was 1.86 times more likely to be incorrectly predicted by the model if it contained less than 170 characters (odds ratio [OR] 1.86; 95% CI 1.20-2.86). Therefore, the significance obtained using these 2 analyses (Mann-Whitney *U* test and OR) allows us to rigorously validate [28] our hypothesis.

### Long Tweet Test

The finding of the previous section is further supported after carrying out the following experiment. Tweets with more than 170 characters were selected from the 400-tweet data set. Classification model 2 was then tested with these 168 tweets to see if its accuracy increased.

As shown in Table 6, the accuracy improved from 64.5% to 73.2% (an 8.7% increase), confirming our hypothesis. The F1-score also increased by approximately the same amount.

**Figure 5:**
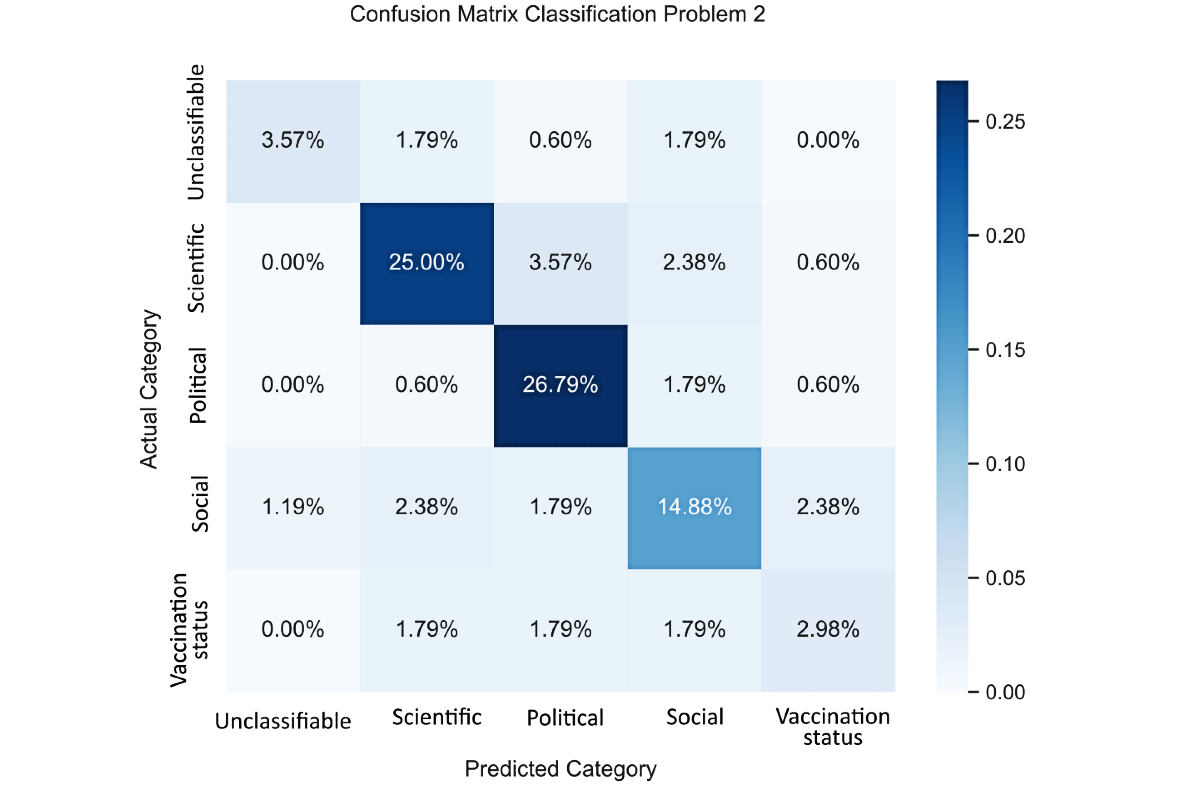
Confusion matrix for classification problem 2 limited to long tweets (n=168).

The confusion matrix generated from the comparison between the model-classified and the manually classified 168 long tweets is shown in Figure 5. From this matrix, it is possible to compute the percentage of correct classifications for each label, the results of which are shown in Table 7. The increase in accuracy is significant for the vaccination status label (an increase of 9.2%), followed by the political label (an increase of 7.7%) and the unclassifiable label (an increase of 6%).

As already pointed out using the Mann-Whitney *U* test and OR, the model for the second problem has much better classification performance with long tweets. It should be noted that the rate of correct classification of political tweets reached an impressive 90% (45/50).

**Table 6:** Classification performance of the model for classification problem 2, limited to long tweets (170 or more characters).

Classification problem	Precision^a^	Recall^a^	F1-score^a^
2	72.6	73.2	72.4

^a^These data are provided as percentages.

**Table 7:** The proportion of correct classifications for each label in classification problem 2, limited to long tweets (170 or more characters; n=168).

Type of problem		Number of tweets
**Classification problem 2, n (%)**		
	Unclassifiable	6 (46.3)
	Scientific	42 (79.2)
	Political	45 (90)
	Social	25 (65.8)
	Vaccination status	5 (35.7)

## Discussion

### Principal Findings

A total of 2 types of classification were examined. The accuracy of the model was better with the second classification (67.6%; F1-score 62.9%) than the first classification (59%; F1-score 55.3%). This accuracy is slightly higher than that obtained by BERT for the same topic (vaccines) [17] and in the same range as previous findings [16,29]. However, CamemBERT obtained a better accuracy (78.7%-87.8%) in a study using dichotomous labels for tweets about eating disorders and using a preprocessing step, reducing the initial number of tweets by 2 [26]. However, by limiting the analysis to long tweets (170 or more characters, in accordance with the statistical analysis conducted on the performance of the model), the accuracy of classification model 2 improved significantly (from 62.9% to 72.4% for the F1-score).

Therefore, as shown by Kummervold et al [17], the classification choices have a significant influence on the accuracy of a model. As in other research areas, the vaccine hesitancy debate crystallizes the opposition. Individuals from the pro and con sides debated on Twitter after the announcement of the implementation of a health pass in July 2021 by the French president. The mobilized arguments were scientific or pseudoscientific to justify or contest this political decision. Several Twitter users participated in the debate to convince anti-vaccine proponents to become vaccinated. Another group of users participated by joking about or ironizing the positions of each side.

Consequently, tweet content is so varied that it remains difficult to manually categorize, and this has been reflected in the model predictions. On the one hand, considering classification problem 1, tweets containing characteristic terms of the anti-vaccine position, such as “5G,” “freedom,” “phase of testing,” “side effect,” and “#passdelahonte” (“shameful pass”), were found to be easier to label and predict. However, because antivaccine proponents spread disinformation more widely on social media [30], the position of provaccine individuals is less polarized [7], which reduces the model’s precision because the terms are less singular. On the other hand, considering classification problem 2, the classes were more distinctive since their lexical fields did not overlap. Indeed, when Twitter users commented on political decisions, the terminology used was different from that used to mobilize scientific or pseudoscientific arguments. Moreover, the scientific and political labels were best predicted by the model (67/84, 79.8% and 93/113, 82.3%, respectively).

Finally, relevant tweets for a topic may be rare in a data set. In some studies, the corpus is halved [13], while in others, only 0.5% (4000/810,600) of downloaded tweets were included in the analysis [16]. It would be interesting to find an objective method to improve model predictions without drastically reducing the data set. The approach of limiting tweet length can be an option, as we have demonstrated in this paper.

### Limitations

Several limitations can be highlighted, including the following: (1) the data were only provided from a single social media platform (Twitter); (2) all tweets containing the term “vaccine” and its derivatives were included without preselection; (3) several categorization classes were unbalanced; (4) a larger training set could provide contrasting results; (5) the categorization choices could affect the performance of CamemBERT, as seen in the confusion matrix; and (6) the suggestions provided (limiting the number of tweet characters) may only apply to tweets on the topic of vaccination, so further studies are needed to confirm the relevance of our conclusions.

### Conclusions

In this study, we tested the accuracy of a model (CamemBERT) without preselecting tweets, and we elaborated an epistemological reflection for future research. When the vaccine debate is jostled by contested political decisions, tweet content becomes so heterogeneous that the accuracy of the model decreases for the less differentiating classes. In summary, our analysis shows that epistemological choices (types of classes) can affect the accuracy of machine learning models. However, our tests also showed that it is possible to improve the model accuracy by using an objective method based on tweet length selection. Other possible avenues for improvement remain to be tested, such as the addition of features provided by Twitter (conservation ID, number of Twitter users following or followers, user public metrics listed count, user public metrics tweet count, or user ID).

## Appendix

Multimedia Appendix 1 Examples of conflicting labeling.

## Abbreviations

API: application programming interface
BERT: Bidirectional encoder representations from transformers
NLP: natural language processing
OR: odds ratio
