# Supplementary material for: An Analysis of French-Language Tweets About COVID-19 Vaccines: Supervised Learning Approach
Source: JMIR Med Inform. 2022 May 17;10(5):e37831. doi: 10.2196/37831 (PMC9116457; doi:10.2196/37831)
Supplement: Multimedia Appendix 1 [file medinform_v10i5e37831_app1.pdf]

Multimedia Appendix 1

Examples of conflicting labeling

4.7% of tweets (87/1851) have been conflictual and needed to be discussed. In accordance with Twitter's terms of use under the European General Data Protection Regulation, these original tweets cannot be shared. However, several types of problems during labeling have been identified and are presented below with modified and selected examples to ensure the anonymity of Twitter users.

Type 1 : the tweets lack information to identify the label

It is a common type encountered. Lack of information conducted us, after verification, to label tweets as unclassifiable or noncommittal.

Example 1:

If the vaccine is so effective why not vaccinate only person's risk of severe illness.

Example 2:

A scary vaccine! So the variants do not scare you!

Type 2 : the "cons" arguments unidentified by one of the two labelers

It is a common type encountered. The sharing of knowledge on the subject and the verification of the labeling criteria allowed these tweets to be classified.

Example 3: freedom is a strong argument among people against vaccination or health measures

Liberty! We are against blackmail, threats, restrictions on our private life.

Example 4: minimizing the effects of COVID and the request of treatment is a strong argument among people against vaccination or health measures.

No one is asking for a vaccine. We demand treatment. Let's stop saying that the virus kills: the vast majority do well.

Example 5: the spread of the virus among vaccinated people is a strong argument among people against vaccination or health measures

The vaccine does not prevent transmission of the virus.

Type 3 : the “pro” arguments unidentified by one of the two labelers

The sharing of knowledge on the subject and the verification of the labeling criteria allowed these tweets to be classified.

Example 6: arguments in favor of vaccination, but which is close to the arguments used by the “cons”

But the virus has not been eradicated and continues to spread with a mutation’s risk. for less than 80% of vaccinated people.

Type 4 : tweets recalling the measures taken during the announcement of the President of the French Republic (health pass, vaccination obligation) or commenting on what the French people are doing.

This type of tweets conducted us, after verification, to label tweets as unclassifiable or noncommital

Example 7:

Will need the vaccine from August to travel by plane, train and bus.

Example 8:

Reinforced health pass, but the young people will obtain special conditions.
